# Detection method of intraoperative awareness: a randomized comparative study

**DOI:** 10.55730/1300-0144.5548

**Published:** 2022-09-12

**Authors:** Meltem BEKTAŞ, Türkay ÇAKAN, Pınar KIRDEMİR, Melis ENGİN, Hülya BAŞAR

**Affiliations:** 1 Department of Anesthesiology and Reanimation, Ankara Training and Research Hospital, Ankara, Turkey; 2 Department of Anesthesiology and Reanimation, Etlik Zübeyde Hanım Women’s Health Training and Research Hospital, Ankara, Turkey

**Keywords:** Brice questionnaire, intraoperative awareness, general anesthesia

## Abstract

**Background/aim:**

The incidence of intraoperative awareness varies in a wide range in the literature. The reasons for these different results include the questioning method used and the questioning time. The goal of this study is to compare the effectiveness of different questioning methods and times used in intraoperative awareness research for detecting the incidence.

**Materials and methods:**

We recruited patients between the ages of 18–70 years, with normal cognitive functions and able to speak after general anesthesia to the study. The patients were randomly divided into two groups. In Group 1 we applied the modified Brice questionnaire in the first 2 h and 24 h after surgery for investigating intraoperative awareness. In Group 2, 24 h after surgery, we asked about anesthesia satisfaction and patients’ complaints, if any.

**Results:**

There was no statistically significant difference between the groups in terms of age (p = 0.514).The proportion of women was significantly higher (p = 0.002), the duration of anesthesia was shorter, and the rate of narcotic analgesic use was higher in Group 2 (p < 0.001). The assessment in the first 2 h showed the frequency of awareness was statistically higher in Group 1 than in Group 2 (p = 0.016). In the postoperative 24-h assessment, we found no significant difference in the incidence of intraoperative awareness between the groups (p < 0.05). In Group 1, there was no statistically significant difference in terms of incidence of awareness according to evaluation time (p = 250).

**Conclusion:**

The incidence of intraoperative awareness in Group 1 was significantly higher than in Group 2 in the evaluation conducted in the first 2 h. There was no significant difference in the determination of intraoperative awareness between questioning times in group 1.

## 1. Introduction

Intraoperative awareness is a complication of general anesthesia and can be defined as a patient’s remembering intraoperative events and expressing this condition [1]. The patient can report the recalled events spontaneously either immediately after the anesthesia experience or later, or the events can be revealed by asking the patient guiding/stimulating questions [2]. It is difficult to determine the intraoperative awareness ratio with certainty, the incidence of awareness during anesthesia varies over a wide range (0.017%–4%) in the studies [3]. The questioning method and the questioning time seems especially important in the determination of incidence [4].

In a study, Mashour compared modified Brice and quality assurance questioning and found that the incidence of awareness was 5 times higher in modified Brice questioning [5]. In modified Brice questioning, the gold standard of evaluation of intraoperative awareness in prospective studies, 2 or 3 interviews were conducted with the participating patients and 6 questions were asked about their anesthesia practice. Quality assurance questioning was performed at the postoperative 24^th^ h and asked the patients if they were satisfied with anesthesia and if they were not, their complaints were learned.

To take correct results regarding intraoperative awareness incidence in our clinics we took Mashour’s study as an example and planned the study.

The primary aim of this study is to determine the incidence of intraoperative awareness in patients who underwent general anesthesia in our clinic. For this purpose, we used different questioning methods and questioning times to decide the correct method and evaluation time. To reveal the causes of awareness, and contribute to the reduction of intraoperative awareness which is a serious source of stress for patients and anesthesiologists, are our secondary goals.

## 2. Materials and methods

This prospective randomized study was conducted in accordance with the Helsinki Declaration and the Good Clinical Practice Directive after receiving the local ethics committee approval and the patients’ written informed consent.

Two thousand one hundred twelve patients aged between 18–70 years, ASA physical status I-III, with normal cognitive functions, planned for surgery under general anesthesia and extubated after an operation and able to speak were included in the study. Outpatient surgeries, cardiac surgeries, patients for whom regional anesthesia was performed, and who were not extubated after surgery or followed up in the intensive care unit were excluded from the study.

Patients were randomly divided into two groups by using computer-generated random numbers in sealed envelopes. Propofol 2mg/kg, fentanyl 1–2μg/kg, rocuronium bromide 0.6 mg/kg were used in the induction of anesthesia and sevoflurane or desflurane with N2O/O2 were used in the maintenance of anesthesia to all the patients. If necessary (in case of hypertension, tachycardia, the necessity of controlled hypotension, etc.), remifentanil infusion with a rate of 0.02–0.2μg/kg/min was given. At the end of the operation after given sugammadex 2mg/kg patients were extubated and transferred to the postanesthesia care unit. Intraoperative awareness was investigated in Group 1 (n = 1086) by applying the modified Brice questionnaire in the first two postoperative h in the recovery room (Brice A) and 24 h later (Brice B) [6]. Modified Brice Questionnaire: 1-What is the last thing you remember before surgery? 2-What is the first thing you remember after recovery? 3-Do you remember anything between the onset of anesthesia and recovery? 4-Did you have a dream during surgery? 5-What was the most unpleasant thing about surgery? 6-Have you had problems falling asleep or during recovery?

Patients in Group 2 (n = 1026) were visited after 24 h postoperatively in accordance with the quality control and assurance in anesthesia and asked whether they were satisfied with anesthesia and if they were not, their complaints were learned (Figure).

Taking into account the patients’ responses to the Brice questioning, whether they experienced awareness was evaluated by a committee of 3 anesthesiologists. The committee used the Michigan awareness classification to determine the awareness [7]. Michigan Awareness Classification: Class 0: no awareness; Class 1: isolated auditory perceptions; Class 2: tactile perceptions (e.g., endotracheal tube or surgical manipulation); Class 3: pain; Class 4: paralysis (e.g., feeling one cannot move, speak, or breathe); Class 5: paralysis and pain.

The data obtained from both groups were evaluated, and the incidence of awareness and the effectiveness of the method used in determining the incidence were evaluated statistically. The answer of Brice A and Brice B were compared to determine whether patients’ awareness changed over time.

Patients characteristics (type of operation, narcotic analgesic usage, etc.) who experienced awareness were also evaluated and tried to find out their effects on awareness.

### 2.1. Statistical analysis

Sample size calculations were made in the G*Power 3.1.9.6 package program (Franz Faul, Universität Kiel, Kiel, Germany). To be able to test the statistical significance of a difference of at least 12% in terms of dissatisfaction with the procedure or awareness of the procedure performed between the groups, at the level of 90% power and 5% error, it was envisaged to include at least 2026 (1013 cases in each of the groups).

The data were analyzed in IBM SPSS Statistics 25.0 (IBM Corporation, Armonk, NY, USA) package program. Whether the discrete numerical variables were normally distributed was examined by the Kolmogorov-Smirnov test. Descriptive statistics are expressed as mean ± standard deviation or median (minimum-maximum) for discrete numerical variables, while categorical variables are shown as the number and percentage (%) of cases. The significance of the difference between the groups in terms of mean age was evaluated with the Student’s t-test, and whether there was a significant difference in terms of anesthesia duration was assessed with the Mann-Whitney U test. The categorical variables were examined by Pearson’s χ^2^ or Fisher’s exact test. The McNemar test was employed to investigate whether there was a statistically significant change in awareness rates for Brice A and Brice B in Group 1. The results for p < 0.05 were considered statistically significant.

## 3. Results

Of the 2112 patients who met the inclusion criteria, Modified Brice questioning was applied to the 1086 patients in the first two postoperative h and 24 h later postoperatively (Group 1), while 1026 patients visited for anesthesia quality control and assurance questioning after 24 h postoperatively (Group 2). The demographic and clinical characteristics of the patients are presented in Table 1.

There was a statistically significant difference between the groups in terms of sex distribution (p = 0.002). The proportion of women was higher and the proportion of men was lower in Group 2 than in Group 1. At the same time, the duration of anesthesia was significantly shorter in Group 2 than in Group 1, while the rate of narcotic analgesic usage was significantly higher (p < 0.001).

According to the Brice A evaluation, 11 (1.0%) cases in Group 1, expressed that they remembered something during the procedure. The evaluation committee decided that 4 out of 11 cases had no awareness. As a result, 7 patients (0.6%) in Group 1, were found to have intraoperative awareness in the assessment conducted in the first 2 h.

In Table 2, intraoperative awareness and satisfaction status of the cases according to the groups are given (Table 2).

There was no statistically significant difference between Group 1 and 2 in terms of awareness of male and female sexes (p = 0.258 and p = 0.055), when all the cases are examined, the frequency of awareness was found to be significantly higher in Group 1 compared to Group 2 (p = 0.016).

In Group 1 Brice B evaluation revealed that, 4 (0.4%) cases, reported experiencing awareness. In Group 2, 4 female patients who expressed dissatisfaction with anesthesia were patients who underwent laparoscopic cholecystectomy surgery. All patients stated nausea and vomiting as the cause of anesthesia dissatisfaction. None of the 4 patients who expressed dissatisfaction had signs of intraoperative awareness. According to the present results, there was no statistically significant difference between the Group 1 and 2 in terms of the frequency of awareness (p > 0.05).

Finally, Table 3, was a 2 × 2 cross tabulation that showed intraoperative awareness prevalence of Group 1. We saw the percentage of intraoperative awareness detected patients in the first 2 h also aware or not in the second evaluation performed in postoperative 24 h (Table 3).

No statistically significant difference was observed in Group 1 when men, women and all the cases were observed in terms of determining the incidence of intraoperative awareness according to evaluation time respectively (p > 0.999, p = 0.500, and p = 0.250).

## 4. Discussion

In this prospective randomized study, we applied the modified Brice questionnaire and asked guiding questions to Group 1. The incidence of intraoperative awareness was 0.6% in the first postoperative questioning, but the incidence decreased to 0.4% in the 2^nd^ questioning after 24 h. In Group 2, we asked anesthesia satisfaction, and we did not detect any intraoperative awareness in any patient.

Determining the incidence of intraoperative awareness with certainty was difficult and the incidence depending largely on the research method was stated before [5].

Mashour compared the modified Brice and the quality assurance questioning and found the intraoperative awareness incidence as 0.1% in the modified Brice questioning and 0.02% in the quality assurance questioning [5]. Similarly, studies using modified Brice as a research method have reported that the intraoperative awareness ratio changes between 0.12%–52% [8,9]. The results of our study revealed similarities with the literature in cases where Brice questioning was applied. The incidence of intraoperative awareness with spontaneous complaints was found to be 0.017% in NAP5 [10]. Pollard found the incidence of awareness as 0.0068% by using quality assurance questions and nonguiding questions [11]. Differently from their findings, we did not detect intraoperative awareness in any of our patients in Group 2. In our study, we had limited case numbers compared to the mentioned studies, which was a possible reason why we have never detected intraoperative awareness in this group of patients.

In the present study, two interviews were conducted with patients; intraoperative awareness was at the rate of 0.6% in the 1^st^ interview and 0.4% in the 2^nd^ interview. Some researchers have claimed that the probability of awareness detection increases with an early interview and decreases with a late interview [12,13]. Conducting multiple interviews with an interval of several days also increases the likelihood of deciphering awareness. Sandin et al. reported that patients experienced awareness at the 2^nd^ or 3^rd^ interview (postoperative 1–3 and postoperative 7–14 days) [14]. In our study, unlike the findings of Sandin, the incidence of awareness was higher in the 1^st^, but lower in the 2^nd^ interview after 24 h. However, this difference was not statistically significant (p > 0.05).

Some studies stated that being female is a risk factor for intraoperative awareness [15,16]. While others claim that intraoperative awareness is independent of sex [17]. In our study, there was no statistically significant difference in awareness between males and females and although female sex ratio was high in Group 2, we did not detect any intraoperative awareness case in this group.

Volatile agents have been reported to cause significantly lower intraoperative awareness risk, when the expiratory MAC values of anesthetics should be monitored and maintained as >0.7 MAC [18]. Although we routinely monitored expiratory MAC values of inhalation agents and tried to maintain MAC >0.7 in our clinic, all the patients whom we detected intraoperative awareness were the patients whose inhalation agents were used. In 3 of the 7 cases where intraoperative awareness was observed, the inhalation agent desflurane/air/O_2_+ remifentanil infusion was present. Inhalation agent sevoflurane was given to 4 patients, and 2 of these cases received sevoflurane/air/O_2_+ remifentanil infusion, and the other 2 patients received sevoflurane/N_2_0/O_2_.

There is a higher risk of developing awareness in case of using high-dose fentanyl during operation [19]. Fentanyl 1–2 μg/kg was used during induction of anesthesia in all the patients and remifentanil infusion was used throughout the case in 5 of the 7 patients in whom awareness was detected in the 1^st^ evaluation and in 3 of the 4 cases in the 2^nd^ evaluation. In these cases, the use of narcotic analgesic infusion might be effective in the development of awareness. In our study, narcotic analgesics usage in Group 2 was significantly higher than in Group 1 but we did not detect any intraoperative awareness case in this group enigmatically. The investigation method was anesthesia control and quality assurance in Group 2 and maybe this was the effective factor for not being identified as having intraoperative awareness. Nitrous oxide was used in 2 of the 7 patients, but the use of nitrous oxide did not affect the incidence of awareness [20].

The duration of the operation might be a risk factor for intraoperative awareness and the risk increased with the prolongation of the duration [15,16]. In our study duration of anesthesia in Group 1 was found significantly longer than in Group 2. This might be one of the factors that affects the incidence of intraoperative awareness in Group 1 was significantly higher than in Group 2.

In Group 2, 4 patients expressed dissatisfaction with anesthesia but none of them had signs of intraoperative awareness. Similar to SNAP-1 study we did not find any relationship between intraoperative awareness and anesthetic care and dissatisfaction [8].

The limitations of the study are; a single-centered study, the number of cases was limited compared to similar studies because of the highly restricted elective surgical procedures due to the Covid-19 pandemic period.

## 5. Conclusion

Modified Brice with guiding questions seems superior to anesthesia quality control and assurance evaluation for determining the incidence of intraoperative awareness in the early postoperative period. Application time of Brice questioning does not have any significant effect on determining intraoperative awareness incidence. Further multi-centered studies with more patients need to be done about this subject.

## Figures and Tables

**Figure:**
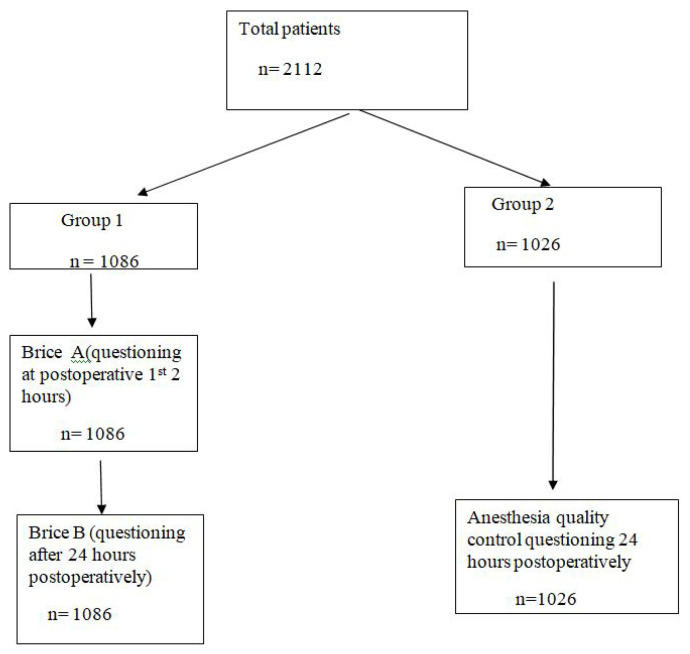
Flow Chart

**Table 1:** Demographic and clinical characteristics of groups.

	Total n = 2112	Group 1 n = 1086	Group 2 n = 1026	p-value
**Age**	45.2 ± 16.5	45.0 ± 16.8	45.5 ± 16.2	0.514†
**Sex**				**0.002** ‡
Male	918(43.5%)	508 (46.8%)	410 (40%)	
Female	1194 (56.5%)	578 (53.2%)	616 (60.0%)	
**Anesthesia duration (min)**	120 (15–480)	120 (20–480)	100 (15–420)	**<0.001** ¶
**Narcotic analgesic usage (μg)**	1556 (73.7%)	736 (67.8%)	736 (67.8%)	**<0.001** ‡

† Student’s t test,

‡ Pearson χ^2^ test,

¶ Mann Whitney U test.

‡,¶ statistically significant

**Table 2:** Intraoperative awareness of the cases according to the groups.

Brice A versus Group 2			
	Group 1	Group 2	p-value†
**Male**			0.258
No awareness	505 (99.4%)	410 (100.0%)	
Awareness	3 (0.6%)	0 (0.0%)	
**Female**			0.055
No awareness	574 (99.4%)	616 (100.0%)	
Awareness	4 (0.6%)	0 (0.0%)	
**Total**			**0.016** *
No awareness	1079 (99.4%)	1026 (100.0%)	
Awareness	7 (0.6%)	0 (0.0%)	
**Brice B versus Group 2**			
	**Group 1**	**Group 2**	**p-value** †
**Male**			0.505
No awareness	506 (99.6%)	410 (100.0%)	
Awareness	2 (0.4%)	0 (0.0%)	
**Female**			0.234
No awareness	576 (99.7%)	616 (100.0%)	
Awareness	2 (0.3%)	0 (0.0%)	
**Total**			0.125
No awareness	1082 (99.6%)	1026 (100.0%)	
Awareness	4 (0.4%)	0 (0.0%)	

† Fisher’s exact test

* statistically significant

**Table 3:** Frequency distributions of the cases in terms of Brice A and Brice B in Group. 1.

**Brice B3**	**Brice A3**				
		No Awareness	Awareness	Total	**p-value** †
	**Male**				>0.999
	No awareness	505 (99.4%)	1 (0.2%)	506 (99.6%)	
	Awareness	0 (0.0%)	2 (0.4%)	2 (0.4%)	
	Total	505 (99.4%)	3 (0.6%)	508 (100.0%)	
	**Female**				0.500
	No awareness	574 (99.4%)	2 (0.3%)	576 (99.7%)	
	Awareness	0 (0.0%)	2 (0.3%)	2 (0.3%)	
	Total	574 (99.4%)	4 (0.6%)	578 (100.0%)	
	**Total**				0.250
	No awareness	1079(99.4%)	3 (0.2%)	1082 (99.6%)	
	Awareness	0 (0.0%)	4 (0.4%)	4 (0.4%)	
	Total	1079(99.4%)	7 (0.6%)	1086(100.0%)	

† McNemar test
